# Transcriptome analysis identifies genes and co-expression networks underlying heat tolerance in pigs

**DOI:** 10.1186/s12863-020-00852-4

**Published:** 2020-04-21

**Authors:** Yuqing He, Christian Maltecca, Francesco Tiezzi, Emmanuel Lozada Soto, William L. Flowers

**Affiliations:** Department of Animal Science, North Carolina State University, Raleigh, NC 27695-7621 USA

**Keywords:** Heat stress, Boar sperm quality, Gene expression, Heat tolerance

## Abstract

**Background:**

Heat stress adversely affects pig growth and reproduction performance by reducing feed intake, weight gain, farrowing rate, and litter size. Heat tolerance is an important characteristic in pigs, allowing them to mitigate the negative effects of heat stress on their physiological activities. Yet, genetic variation and signaling pathways associated with the biological processes of heat-tolerant pigs are currently not fully understood. This study examined differentially expressed genes and constructed gene co-expression networks on mRNAs of pigs under different heat-stress conditions using whole transcriptomic RNA-seq analyses. Semen parameters, including total sperm number per ejaculate, motility, normal morphology rate, droplets, and rejected ejaculate rate, were measured weekly on 12 boars for two time periods: thermoneutral (January to May), and heat stress (July to October). Boars were classified into heat-tolerant (*n* = 6) and heat-susceptible (n = 6) groups based on the variation of their ejaculate parameters across the two periods. RNA was isolated from the blood samples collected from the thermoneutral and heat stress periods for gene expression analysis.

**Results:**

Under heat stress, a total of 66 differentially expressed genes (25 down-regulated, 41 up-regulated) were identified in heat-tolerant pigs compared to themselves during the thermoneutral period. A total of 1041 differentially expressed genes (282 down-regulated, 759 up-regulated) were identified in the comparison between heat-tolerant pigs and heat-susceptible pigs under heat stress. Weighted gene co-expression network analysis detected 4 and 7 modules with genes highly associated (r > 0.50, *p* < 0.05) with semen quality parameters in heat-tolerant and heat-susceptible pigs under the effects of heat stress, respectively.

**Conclusion:**

This study utilized the sensitivity of semen to heat stress to discriminate the heat-tolerance ability of pigs. The gene expression profiles under the thermoneutral and heat stress conditions were documented in heat-tolerant and heat-susceptible boars. Findings contribute to the understanding of genes and biological mechanisms related to heat stress response in pigs and provide potential biomarkers for future investigations on the reproductive performance of pigs.

## Background

As global temperatures continue to increase, the swine industry faces severe challenges related to heat stress (**HS**). Being one of the major environmental challenges on agriculture, HS negatively affects the immune functions, metabolism, fertility, and welfare at every stage of the production cycle [1–3]. High temperature environments present challenges for animals’ health as the suppression of the immune system leads to an increase in the occurrence of disease [4]. Additionally, in pigs, HS negatively impacts productivity through increasing the whole-body insulin sensitivity and preventing adipose tissue mobilization [5].

It has been estimated that in the United States economic losses in the swine industry due to HS have reached one billion dollars per year in recent years [6] . Pigs are highly susceptible to increased environmental temperatures given a lack of effective sweat glands for thermoregulation and the presence of a thick layer of subcutaneous adipose tissue which reduces the heat exchange capacity [3]. Additionally, intense genetic selection for production traits has contributed to a reduction in heat tolerance due to the large amount of metabolic heat generated by higher producing animals [6–8].

Reduction in the fertility of boars due to HS has become an increasing threat to the profitability of the industry. Seasonal infertility due to elevated temperature of domestic boars has been reported between June to November in many countries around the world [2]. Semen quality, as a representative index for fertility in pig reproduction, is well known to be highly susceptible to environmental stressors. Significant reductions in sperm production, semen quality and fertility have been documented in boars exposed to a period of elevated ambient temperatures [9, 10] . Good management strategies can buffer part of the negative influences of elevated temperatures on boars. However, even under proper management, a significant reduction in semen quality, in terms of semen concentration, sperm motility, the percentage of sperm with normal morphology, and the ejaculate rate, can still be observed in boars when temperatures exceed 27 °C, which is considered as the upper limit of the thermoneutral (**TN**) zone for adult swine [9, 11, 12].

The physiological response to high temperatures varies between individuals, with some pigs showing higher tolerance to increased temperature and humidity. In commercial boars, exposure to an average daily temperature of 24 °C and three time point of maximum temperature exceeding 27 °C was found to affect boars differently, with the majority of boars experiencing decline in sperm viability and some experiencing marginal to null decline in this parameter [13]. Due to this variability in thermotolerance, selection for more adaptable pigs has become a strategy in urgent need of implementation, especially in countries with tropical environments.

Although phenotypic variation in heat-tolerant pigs in terms of reproductive performance has been documented [14, 15], no gene expression profiling studies to the best of our knowledge have been performed to compare them to heat-susceptible pigs. Understanding the gene expression pattern behind phenotypic variation to HS could provide new insights about the biological mechanisms discriminating heat-tolerant from heat-susceptible pigs. Benefiting from the recent advantages in differential gene expression analysis and genetic network construction approaches, in this study we employed a transcriptomic approach to quantify gene expression in blood samples of pigs classified as tolerant or susceptible under HS condition [16]. To investigate the whole-body homeostatic mechanisms under the effects of HS [17], blood could be a good target sample to profile the expression of genes associated with the physiological responses induced underlying HS [18]. Moreover, we performed a gene co-expression network analysis [19], to detect functional modules of genes highly associated with semen quality characteristics in the two groups.

## Results

### Semen quality parameters and group classification

In this study, we utilized the change of semen quality parameters due to HS to distinguish heat-tolerant pigs from the population to document gene expression patterns of heat-tolerant and heat-susceptible pigs under TN and HS conditions. Average temperatures during the TN and HS data collection periods in 2016 and 2017 were similar, which was about 11 °C during the TN period and 26 °C during the HS period.

Semen quality parameters collected in 2016 were statistically compared between TN and HS periods to classify boars into heat-tolerant and heat-susceptible groups. The parameters included total sperm number per ejaculate, sperm motility, normal morphology rate, cytoplasmic droplets, and rejected ejaculated rate measured, total sperm number per ejaculate and rejected ejaculated rate. For the heat-tolerant group, no significant changes in total sperm number per ejaculate (mean: 72.8 billion vs. 83.9 billion, *P* = 0.25) and rejected ejaculated rate (mean: 3.6% vs. 1.4%, *P* = 0.64) were observed between TN and HS periods. However, for the boars in the heat-susceptible group, total sperm number per ejaculate decreased from 82.1 to 64.7 billion (*P* < 0.001) and rejected ejaculated rate increased from 11 to 27% (*P* = 0.01) during the HS period compared to the TN period. Descriptive statistics of semen quality parameters are presented in Table 1.

**Table 1:** Summary statistics for semen quality parameters of boars measured in TN and HS periods (2016)

Phenotype	Period	Statistics	Semen Quality Parameters^**1**^				
			Total Sperm (billion)	Motility (%)	Normal Morphology (%)	Droplets (%)	Rejected Ejaculate (%)
Tolerant (n = 6)	TN	Mean ± SD	72.9 ± 6.3^a^	88.0 ± 4.0^a^	90.1 ± 4.5^a^	2.5 ± 1.0^a^	3.7 ± 4.9^a^
		Range	63.1–79.4	83.3–92.6	84.4–95.2	1.3–3.8	0.0–10.4
	HS	Mean ± SD	83.9 ± 7.2^a^	86.3 ± 6.1^a^	90.2 ± 4.1^a^	2.9 ± 1.0^a^	1.4 ± 3.3^a^
		Range	75.4–94.3	78.5–93.1	83.5–94.1	2.0–4.1	0.0–8.2
Susceptible (n = 6)	TN	Mean ± SD	82.1 ± 6.8a	91.2 ± 2.3^a^	86.0 ± 4.8^a^	6.6 ± 7.5^a^	11.3 ± 6.6^a^
		Range	71.3–89.4	88.3–94.3	78.2–91.2	0.8–21.4	1.4–21.5
	HS	Mean ± SD	64.7 ± 11.8^b^	78.5 ± 2.7^a^	79.0 ± 10.6^a^	12.6 ± 11.5^a^	27.3 ± 10.5^b^
		Range	44.2–78.3	74.2–82.2	63.4–92.3	1.8–32.9	15.3–46.8

^1^Means followed by different letters are significantly different (*P* < 0.05)

Using the previous classification scheme, the semen quality parameters and blood samples were collected from the same boars in 2017 for gene expression analysis. The total sperm number per ejaculate and rejected ejaculated rate showed a similar trend as what we observed in 2016. In the heat-tolerant group, the total sperm number per ejaculate increased from 69.9 to 86.3 billion from the TN to the HS period (*P* = 0.01) and the rejected ejaculated rate did not significantly change with 1.3 and 5.8% during the TN and HS period, respectively (*P* = 0.36). In contrast, in the heat-susceptible group, the total sperm number per ejaculate decreased from 87.5 billion in TN period to 71 billion in HS period (*P* = 0.01). Similarly, the rejected ejaculated rate increased from 23% in TN period to 32% in HS period (*P* = 0.05). Descriptive statistics of semen quality parameters are included in Table 2.

**Table 2:** Summary statistics for semen quality parameters of boars measured in TN and HS periods (2017)

Phenotype	Period	Statistics	Semen Quality Parameters^**1**^				
			Total Sperm (billion)	Motility (%)	Normal Morphology (%)	Droplets (%)	Rejected Ejaculate (%)
Tolerant (n = 6)	TN	Mean ± SD	69.9 ± 12.1^a^	88.6 ± 4.5^a^	96.3 ± 0.6^a^	2.5 ± 1.0^a^	5.8 ± 6.8^a^
		Range	53.4–85.9	83.3–93.3	95.6–97.0	1.4–3.6	0.0–15.0
	HS	Mean ± SD	86.3 ± 14.5^b^	89.1 ± 7.5^a^	90.0 ± 3.7^a^	6.1 ± 2.9^a^	1.3 ± 3.3^a^
		Range	67.6–107.6	74.5–95.1	85.0–94.2	2.9–10.3	0.0–8.0
Susceptible (n = 6)	TN	Mean ± SD	87.6 ± 13.7^a^	92.2 ± 2.8^a^	86.5 ± 12.0^a^	8.1 ± 12.2^a^	23.0 ± 23.3^a^
		Range	74.3–111.4	88.6–95.9	63.3–97.6	0.8–32.8	0.0–67.0
	HS	Mean ± SD	71.0 ± 15.8^b^	82.8 ± 2.5^a^	88.9 ± 12.1^a^	9.0 ± 10.9^a^	32.3 ± 24.6^b^
		Range	40.1–81.9	79.2–85.7	73.2–98.0	1.3–24.2	15.0–81.0

^1^Means followed by different letters are significantly different (*P* < 0.05)

### Identification of differentially expressed genes

Transcriptional RNA expression analysis was performed to compare the expression pattern in heat-tolerant vs. heat-susceptible pigs during the TN and HS periods. After removal of genes with low counts, a total of 10,761 genes remained in the gene set for further analysis. The *duplicateCorrelation* function in the *‘limma’* package (v.3.40.6) [20] was used to fit the boar as a random effect in the model to control the potential batch effects because the same boar was measured twice for TN and HS periods. Genes with FDR < 0.05 were considered as DEGs. The log2 fold-change (log2 FC) was used to show a gene’s expression value in terms of log ratio in two different conditions.

**Fig. 1:**
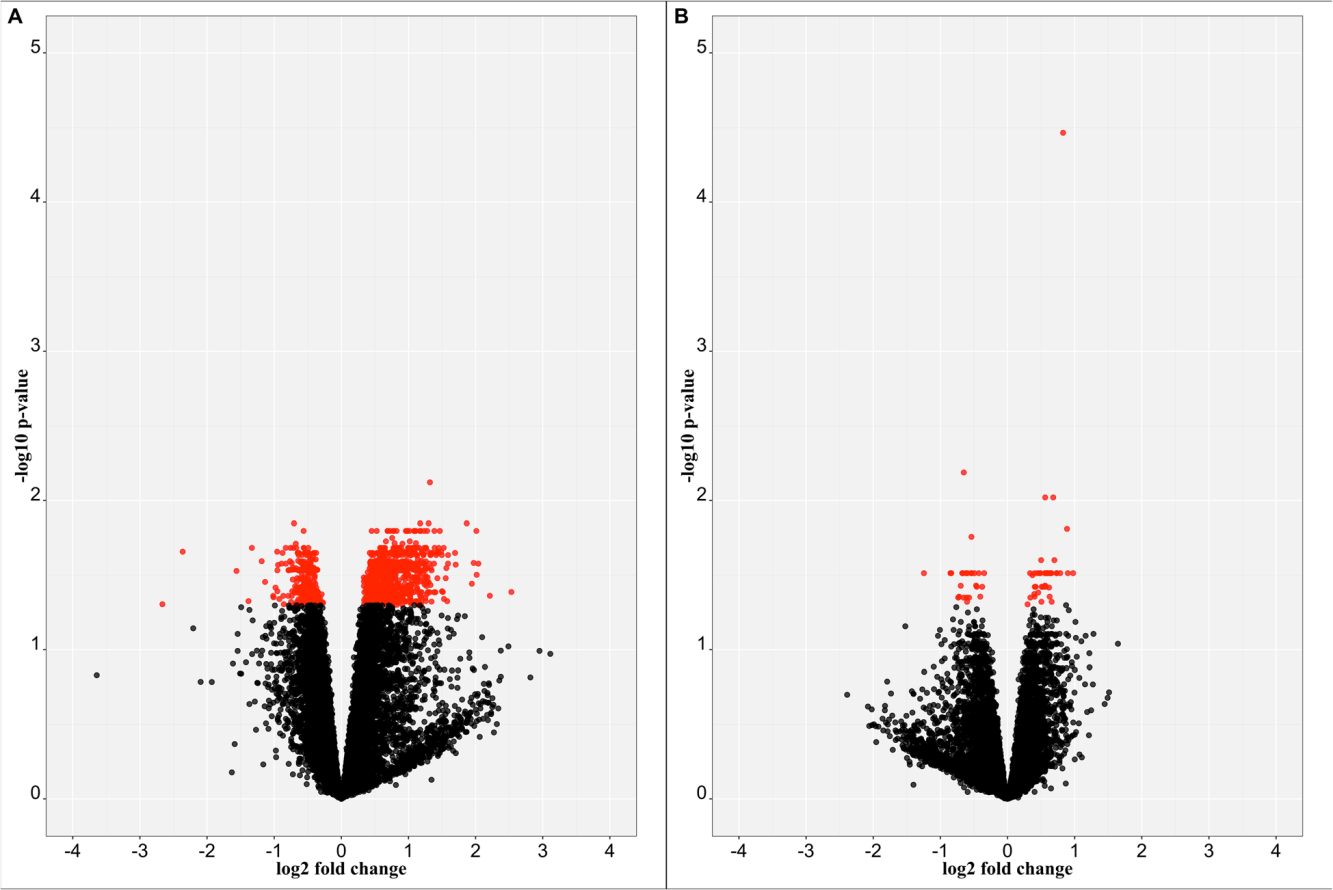
Volcano plot of DEGs. The x-axis represents the log2 FC of gene expression level between two conditions. The y-axis represents the -log10 FDR. Genes with FDR < 0.05 are shown in red points. **a**. Heat-tolerant pigs in HS period versus heat-susceptible pigs in HS period. **b**. Heat-tolerant pigs in HS period versus themselves in TN period

No significant DEGs were found when contrasting heat-tolerant and heat-susceptible pigs during the TN period. Counter to that, when comparing heat-tolerant and heat-susceptible pigs during the HS period, a total of 759 up-regulated and 282 down-regulated DEGs were identified (Fig. 1). Additionally, a total of 41 up-regulated and 25 down-regulated DEGs were identified in heat-tolerant pigs during the HS period compared to themselves during the TN period (Fig. 1), while no DEGs were found in heat-susceptible pigs between the TN and HS periods.

**Fig. 2:**
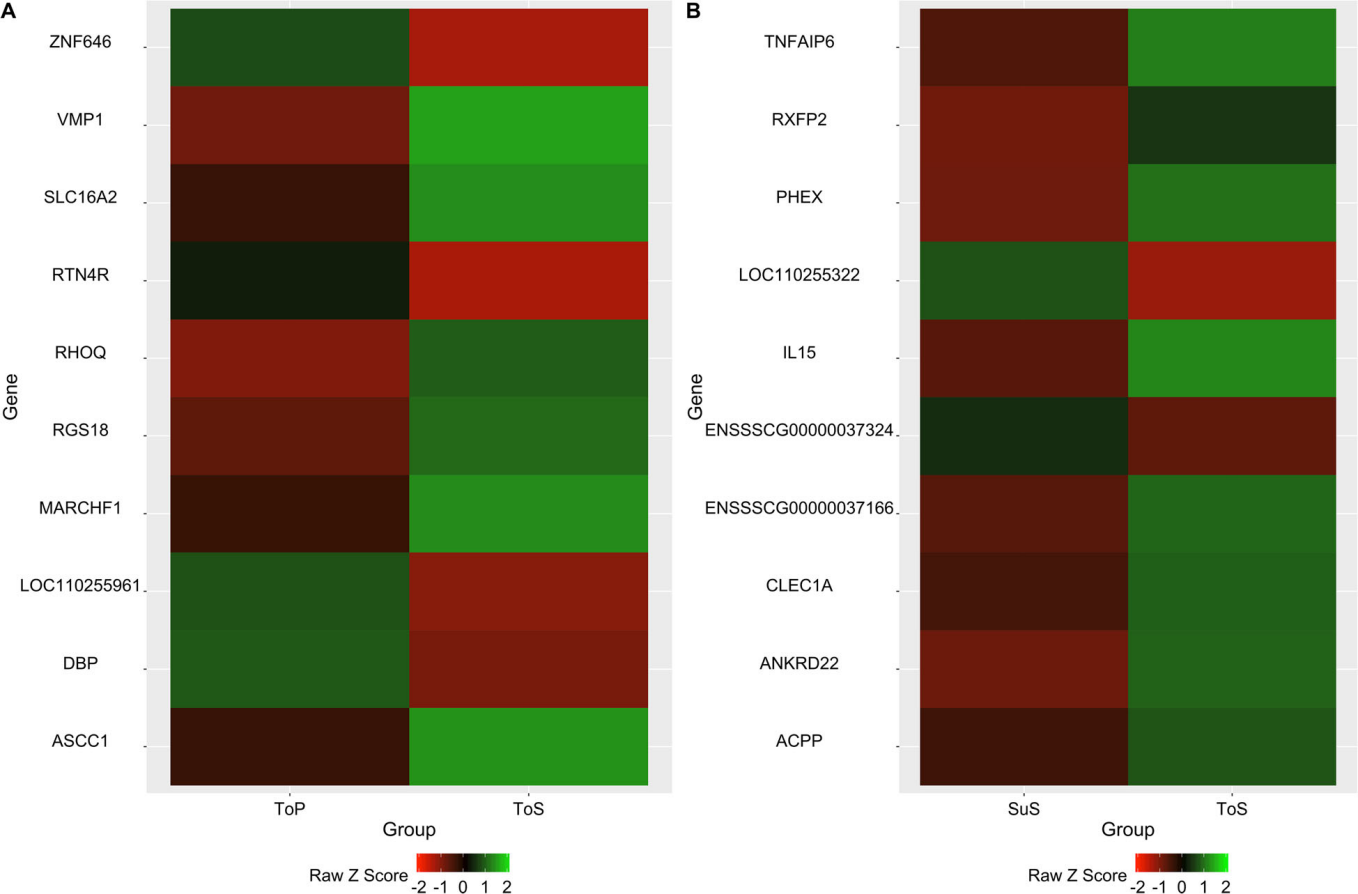
Expression profiles of top 10 DEGs ranked by log2 FC (FDR < 0.05). **a**. Heat-tolerant pigs in HS period vs. heat-tolerant pigs in TN period. **b**. Heat-tolerant pigs in HS period vs. heat-susceptible pigs in HS period

The top 5 up- and down-regulated genes ranked by FDR are listed in Table 3, while the expression profiles of the top 10 DEGs ranked by log2 FC are presented in Fig. 2. The top 5 up-regulated genes found in the contrast of heat-tolerant pigs between the TN and HS periods included RGS18, SLC16A2, MARCHF1, VMP1, and ASCC1. Among them, RGS18, SLC16A2 and MARCHF1 are involved in molecular and cellular signaling and transportation activities. During the HS period the top 5 DEGs between heat-tolerant and heat-susceptible pigs were CLEC1A, TNFAIP6, ACPP, RXFP2, and IL15. Most of these genes are related to the innate immune function and inflammatory response.

**Table 3:** List of the top 5 significantly up- and down- regulated genes

Contrast	Up/Down	Gene ID	Gene Symbol^**1**^	Log2 FC	FDR
Tolerant (HS) vs. Tolerant (TN)	Up	ENSSSCG00000033945	RGS18	0.99	0.0306
		ENSSSCG00000029458	SLC16A2a	0.90	0.0306
		ENSSSCG00000039175	MARCHF1	0.89	0.0154
		ENSSSCG00000017668	VMP1	0.83	3.42E-05
		ENSSSCG00000023130	ASCC1	0.78	0.0306
	Down	ENSSSCG00000034656	RTN4R	−1.25	0.0306
		ENSSSCG00000035507	LOC110255961	−0.85	0.0306
		ENSSSCG00000026817	ZNF646	−0.84	0.0306
		ENSSSCG00000003148	DBP	−0.73	0.0447
		ENSSSCG00000014046	ZNF346	−0.72	0.0440
Tolerant (HS) vs. Susceptible (TN)	Up	ENSSSCG00000000649	CLEC1A	2.53	0.0410
		ENSSSCG00000023716	TNFAIP6	2.21	0.0434
		ENSSSCG00000011627	ACPP	2.04	0.0264
		ENSSSCG00000009336	RXFP2	2.02	0.0314
		ENSSSCG00000009051	IL15	2.01	0.0159
	Down	ENSSSCG00000037324	N/A	−2.67	0.0495
		ENSSSCG00000031085	WC1	−2.36	0.0220
		ENSSSCG00000025784	CDH4	−1.56	0.0296
		ENSSSCG00000011119	ECHDC3	−1.38	0.0472
		ENSSSCG00000034656	RTN4R	−1.33	0.0207

^1^N/A = No symbol name was found

### Gene ontology (GO) enrichment analysis on DEGs

**Fig. 3:**
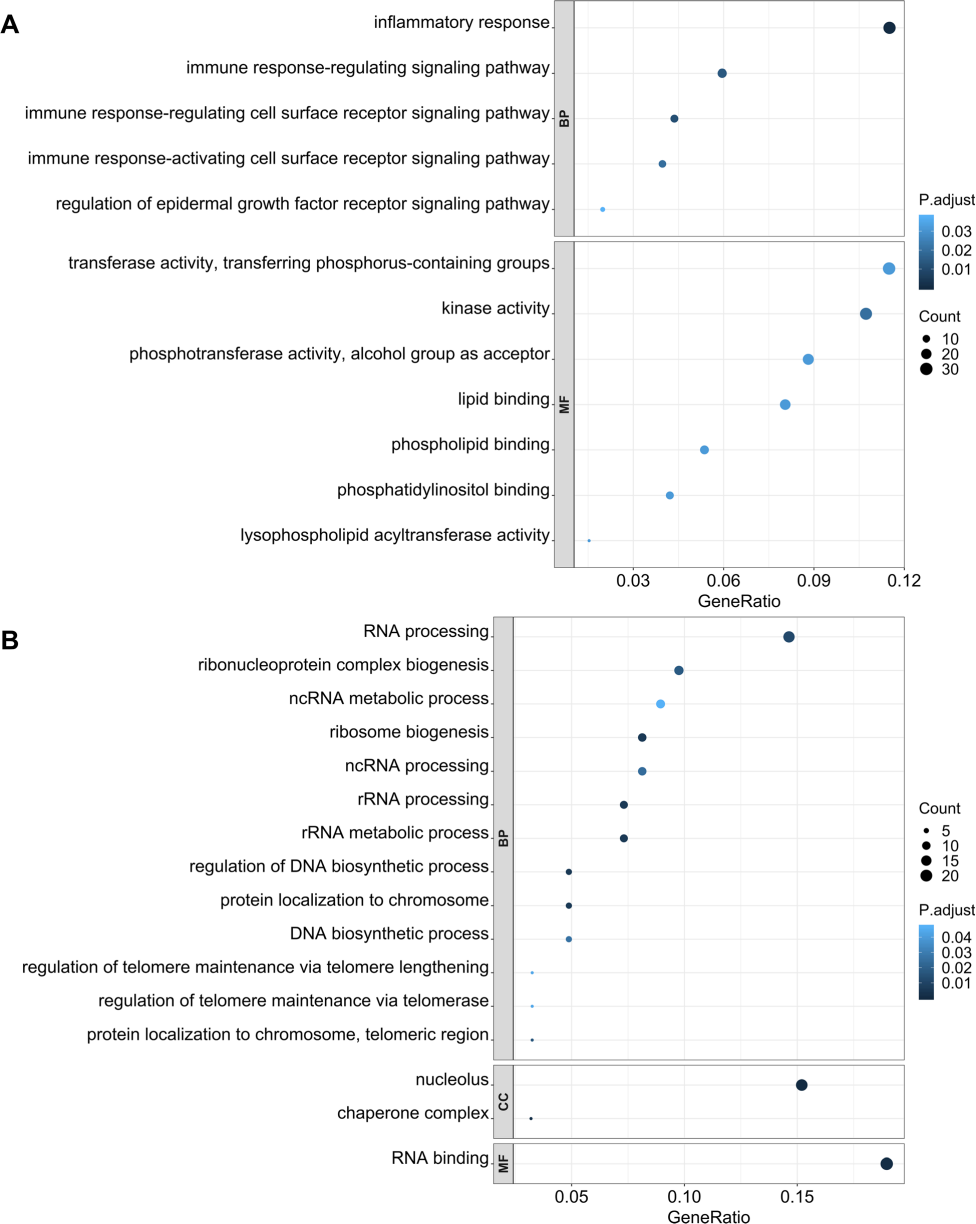
Dot plot of GO analysis on DEGs between heat-tolerant pigs and heat-susceptible pigs under HS. The x-axis represents the number of genes involved in the given GO terms. The y-axis represents the GO terms grouped by GO categories. Color shows FDR. **a**. GO terms of up-regulated DEGs. **b**. GO terms of down-regulated DEGs. Each significant ontology (FDR < 0.05) is represented by a dot

Both up- and down-regulated DEGs were subjected to GO enrichment. Ontology terms of DEGs were presented as three categories: biological process (**BP**), molecular function (**MF**), and cellular component (**CC**). Significant GO terms with gene count and Benjamini-Hochberg adjusted *P*-value are presented in Fig. 3. For the contrast between heat-tolerant pigs under the HS and TN conditions, a total of 18 out of 41 up-regulated DEGs were annotated into CC. Three genes: STX12, RAB8B, SYK, were significantly enriched in two CC groups: *phagocytic vesicle* and *endocytic vesicle*. No significant GO terms were found in the BP and MF categories. A total of 12 out of 25 down-regulated DEGs were annotated, but no significant GO terms were found in these 12 annotated DEGs. When contrasting heat-tolerant and heat-susceptible pigs under HS, inflammatory response and immune response signaling pathways were mainly enriched for the up-regulated DEGs in the BP category. Activities of *transferase*, *kinase*, *phosphotransferase*, *lysophospholipid acyltransferase*, *lipid*, *phospholipid*, and *phosphatidylinositol binding* were enriched for the up-regulated DEGs in the MF category. Mainly RNA and DNA related processes and *biogenesis* were enriched for down-regulated DEGs.

### Gene co-expression network construction and correlation with semen parameters

Gene expression profiles of pigs under HS were examined using weighted gene co-expression network analysis (WGCNA) to detect co-expressed genes associated with semen quality parameters. Modules with highly correlated (r > 0.80) eigengenes were merged and were assigned different colors, with the color name being a module identifier. Identified modules referred by their color labels and the clustering relationships among modules are depicted in the hierarchical clustering dendrograms (Additional file 2).

**Fig. 4:**
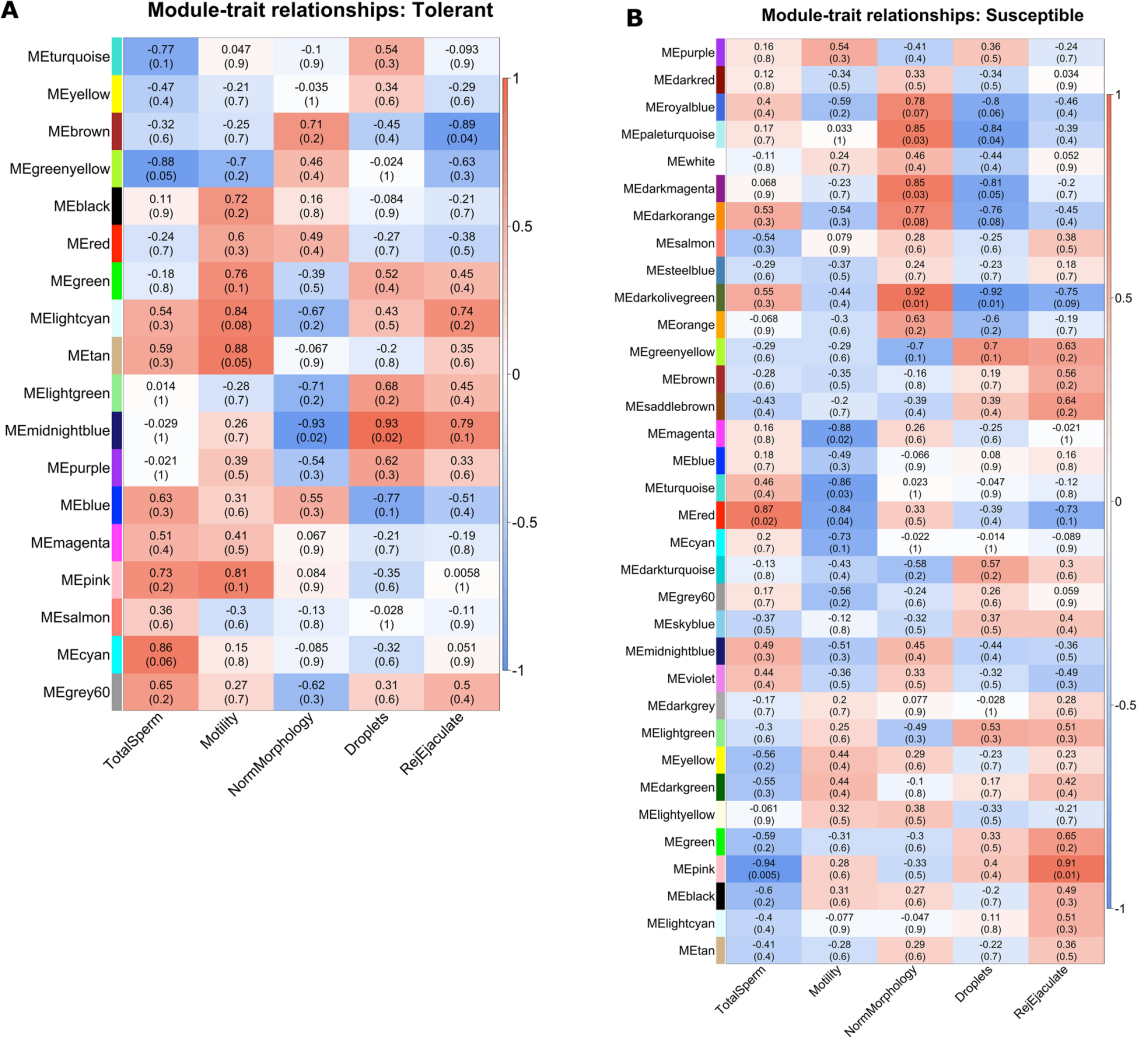
Heatmap of correlations between identified modules of networks and semen quality parameters. Each row corresponds to a module eigengene, column to a trait. Each cell contains the corresponding correlation and (*p*-value). The table is color-coded by correlation according to the color legend. **a**. Heat-tolerant pigs under HS. **b**. Heat-susceptible pigs under HS

Associations between identified modules and semen quality parameters were detected by calculating the correlation between gene expression and the parameters. Four modules were significantly correlated with a semen quality parameter at the defined cut-offs (r > 0.50 and *P*-value < 0.05) in heat-tolerant pigs (Fig. 4). The size of these four modules ranged from 120 to 1234 genes, and 4 to 18% of the genes belonging to the modules were also DEGs between heat-tolerant and heat-susceptible pigs during the HS period. The *greenyellow* module showed negative correlation with number of sperm (*r* = − 0.88, *P* = 0.05). The *tan* module showed positive correlation with motility (*r* = 0.88, *P* = 0.05). The *midnightblue* module was negatively associated with normal morphology rate (*r* = − 0.93, *P* = 0.02), while it was positively associated with droplets (*r* = 0.93, *P* = 0.02). The *brown* module was negatively correlated with rejected ejaculate rate (*r* = − 0.89, *P* = 0.04). In the heat-susceptible group, seven modules were significantly correlated with a semen quality parameter (Fig. 4). The size of these seven modules ranged from 33 to 2380 genes. About 3 to 23% of the genes belonging to the modules were DEGs found between heat-tolerant and heat-susceptible pigs during the HS period. The *red* module was positively correlated to number of sperm (*r* = 0.87, *P* = 0.02), but was negatively associated with motility (*r* = − 0.84, *P* = 0.04) as well as the *magenta* (*r* = − 0.88, *P* = 0.02) and *turquoise* modules (*r* = − 0.86, *P* = 0.03). The *pink* module showed strong negative correlation with number of sperm (*r* = − 0.94, *P* = 0.005) and strong positive correlation with rejected ejaculate rate (*r* = 0.91, *P* = 0.01). The *paleturquoise (r* = 0.85, *P* = 0.03), *darkmagenta (r* = 0.85, *P* = 0.03), and *darkolivegreen* (*r* = 0.92, *P* = 0.01) modules showed strong positive correlations to normal morphology percent, and strong negative correlations to droplets (*paleturquoise*: *r* = − 0.84, *P* = 0.04; *darkmagenta*: *r* = − 0.81, *P* = 0.05; *darkolivegreen*: *r* = − 0.92, *P* = 0.01).

### Relationship between gene significance and module membership

The correlation between the gene’s expression pattern and the module eigengene, known as module membership (**MM**), measures the strength of an individual gene’s membership in a given module. Gene significance (**GS**) describes the biological relevance of the given gene to the phenotypic traits through obtaining the correlation values between them. In both co-expression networks, GS and MM were highly correlated, which means those genes highly associated with the semen quality traits serve important roles in the given module (*P* < 0.05, Table 4). The relationship between the significance level of DEGs derived from the differential gene expression analysis and MMs is depicted in Additional file 3. The descriptive statistics tables for each significant module are also included. As expected, the majority DEGs with very high MM values play a critical role interacting with other genes in the given module.

**Table 4:** Correlations between Gene Significant and Module Membership of selected modules associated with semen parameters

Group	Module	Module size (No. of Genes)	Module Membership vs. Gene Significance	
			Correlation (r^**2**^)	P-value
Heat-tolerant	Greenyellow	168	0.33	<1e-3
	Tan	167	0.40	<1e-5
	Midnightblue	120	0.53	<1e-8
	Brown	1234	0.49	<1e-8
Heat-susceptible	Red	480	0.60	<1e-8
	Pink	250	0.60	<1e-8
	Magenta	199	0.41	<1e-8
	Turquoise	2380	0.63	<1e-8
	Paleturquoise	46	0.63	<1e-5
	Darkmagenta	33	0.44	<1e-3
	Darkolivegreen	38	0.68	<1e-5

### Hub genes and functional analysis on selected modules

**Fig. 5:**
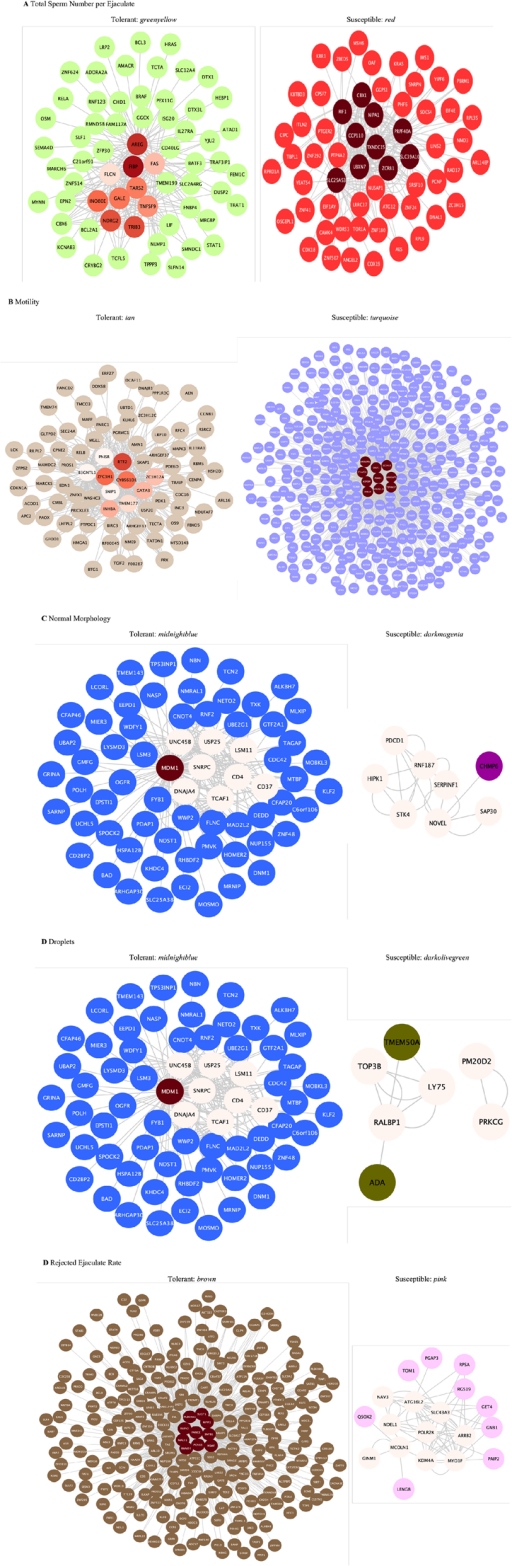
Network of top 10 hub genes and their connections in selected modules. **a**. Total sperm number per ejaculate, **b**. Motility, **c**. Normal morphology rate, **d**. Droplets, **e**. Rejected ejaculate rate parameters in heat-tolerant pigs and heat-susceptible pigs under HS, respectively. Color of hub genes: deeper color indicates higher connectivity values. Color of general genes: corresponding colors of modules

Hub genes are defined as genes with high intra-module connectivity. Intra-module connectivity of each gene in interested modules was computed to examine the connectivity between nodes in a module. Highly connected hub genes within a module can be interpreted as genes playing critical roles in biological processes associated with semen characteristic in pigs challenged by HS. The 10 genes with the greatest intra-module connectivity values in interested modules were selected as candidate hub genes. The networks of hub genes and their connections for each module are shown in Fig. 5. Hub genes within each module significantly associated with semen quality parameters may have the potential as biomarkers for HS in heat-tolerant and heat-susceptible groups.

**Fig. 6:**
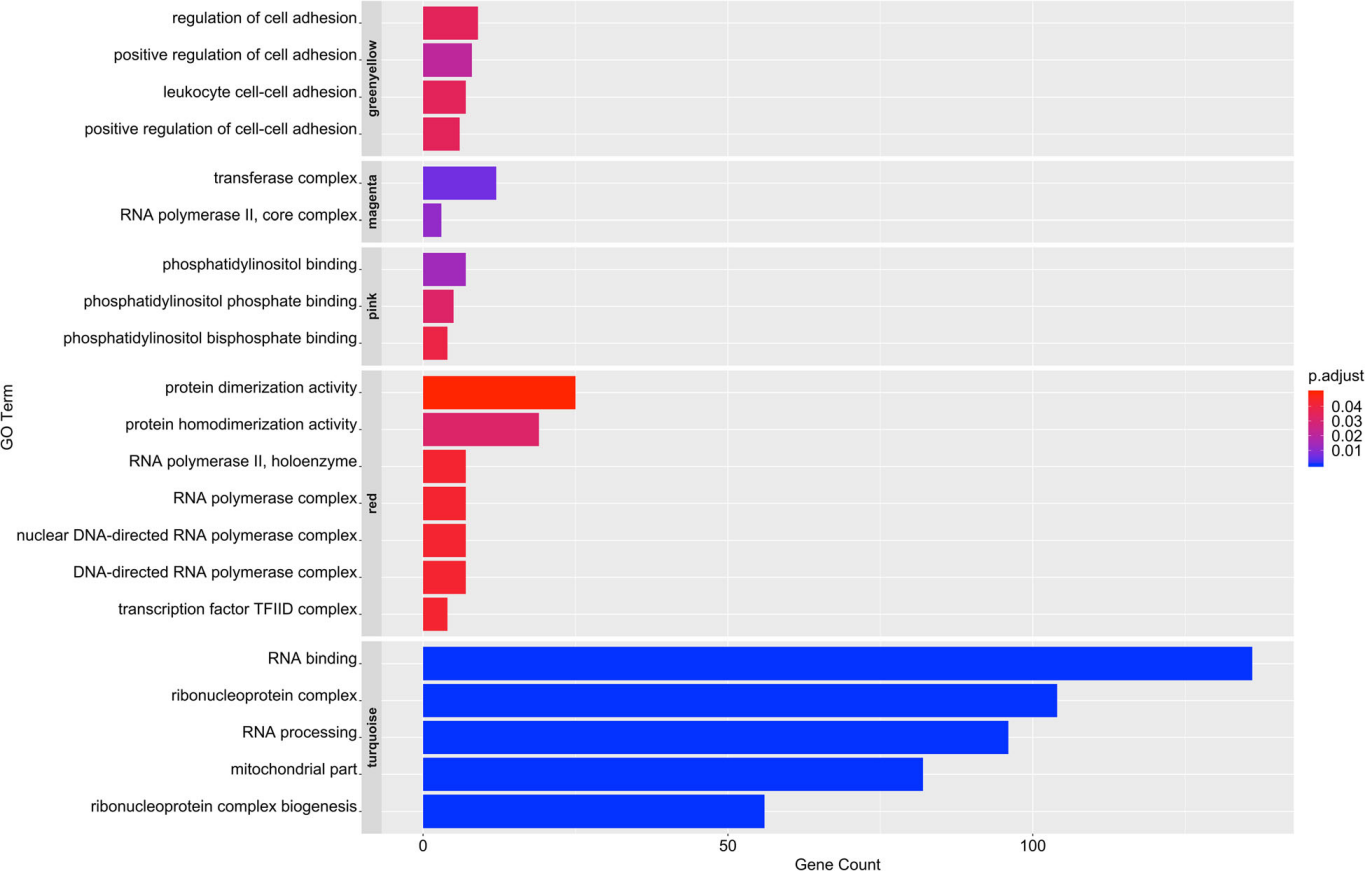
Bar plot of GO analysis on genes within selected modules. The x-axis represents the number of genes involved in GO terms. The y-axis represents the GO terms grouped by modules. Color shows FDR

Gene ontology enrichment analysis was carried out on genes within each selected module. Significant GO terms for each module in either heat-tolerant or heat-susceptible groups are listed in Fig. 6 (FDR < 0.05). For heat-tolerant pigs, one out of four modules was annotated. The genes of the *greenyellow* module were enriched in biological process related cell adhesion. For heat-susceptible pigs, four out of seven modules were enriched. Three modules (*magenta, red*, and *turquoise*) were related to RNA transcription activities and one module (*pink*) was enriched in phosphatidylinositol binding.

## Discussion

Unlike other species, reproduction in boars is not limited to a particular season, therefore the maintenance of adequate sperm parameters year-round is essential. Achieving this can be difficult, owing to the sensitive nature of sperm maturation to elevated and fluctuating temperatures [2]. Significant reductions in semen volume and concentration [15], sperm motility [21], the percentage of morphologically normal sperm [22], and ejaculate rate [11], have been reported with HS. The reduction in semen quality may require more boars to be kept in the breeding system to maintain a normal breeding rate during summertime, which is not cost-effective for the swine industry.

Boars respond to HS differently in terms of semen quality. A study has observed individual variations in response to HS in terms of semen volume, sperm concentration, total sperm number per ejaculate, and sperm motility [15]. Breeds developed around equator showed better heat tolerance than those raised in northern Europe [23]. In our study, the temperature during the HS period exceeded the optimal environmental temperature previously found (18 to 21 °C) to maintain reproductive performance in swine [24]. The balance between heat production and heat loss is broken when exposing pigs to these elevated daily temperatures exceeding their TN zone [24]. But some boars showed tolerance to HS in terms of total sperm number per ejaculate and rejected ejaculate rate (Additional file 4). Total sperm per ejaculate increased in heat-tolerant pigs from the TN to the HS period. This may due to the development of boars and the production of sperm reached the peak during that period. From a physiological and practical perspective, total sperm number per ejaculate and rejected ejaculate rate are the two parameters mostly used to evaluate semen quality. The total sperm number is used to determine insemination doses. An ejaculate can be rejected for having motility or normal morphology lower than 70% or for having cytoplasmic droplets higher than 15 to 20%. In practice, if any of these is met, the ejaculate cannot be used. Therefore, we identified any variation in gene expression associated with biological mechanisms affecting tolerance/susceptibility to HS in pigs.

Among all the DEGs identified in heat-tolerant pigs during the HS period, either compared to themselves or to heat-susceptible pigs, we found some to possibly contribute to heat resistance and immune function activation, mainly SLC16A2, MARCHF1, TNFAIP6, RXFP2, and IL15. The gene SLC16A2 (solute carrier family 16 member 2), serves as an important transporter of thyroid hormones with several biological functions. In our study, SLC16A2 was significantly up-regulated in heat-tolerant pigs under HS compared to TN period (log2 FC = 0.90, *P* = 0.03). Studies have revealed that pigs under HS showed a marked decrease in the levels of thyroid hormones, which might result in the reduction of adipose tissue mobilization and the activation of lipolysis [5, 8]. Thus, the up-regulation of SLC16A2 in heat-tolerant pigs could contribute to HS resistance. The gene VMP1(vacuole membrane protein 1), encoding a transmembrane protein involved in cell autophagy, was also up-regulated in heat-tolerant pigs under HS compared to TN period. Autophagy in oxidative skeletal muscle of pigs has been found to increase during acute HS due to a larger cell apoptosis mediated by HS [25]. The gene IL15 (interleukin 15), expressed in subcutaneous adipose of pigs, has been linked to T-cell growth and development, as well as stimulating lipolysis during acute inflammatory response [26]. The cytokine IL-15 coded by IL15 gene is widely known as a regulator of T cell homeostasis, including the maintenance of naïve and memory T cells [27]. Additionally, IL-15 plays an important role in promoting the survival of the short-lived CD8^+^ T cells, which serve as main effectors in immune defense [28]. To be noticed, the gene RXFP2 (relaxin/insulin-like family peptide receptor 2), mainly expressed in meiotic and post-meiotic testicular germ cells, functions as a survival/antiapoptotic factor in germ cells activated by its specific molecular INSL3 in human reproduction [29]. In boars, RXFP2 is expressed in seminiferous germ cells and it functions with INSL3 as a mediator in maintaining sperm production but it was found expressed in the blood in our study [30, 31]. The results of this study suggest that DEGs up-regulated in heat-tolerant pigs might contribute to the heat resistance and boost immune functions compared to heat-susceptible pigs. The DEGs were functionally annotated based on GO terms, five GO terms were mapped in the BP category for up-regulated genes found in the heat-tolerant pigs compared to the heat-susceptible pigs during the HS period. Corresponding to the functions of DEGs, the majority of the terms related to the regulation and activation of immune and inflammatory responses. The gene RABGEF1 (RAB guanine nucleotide exchange factor1), involved in these immune function related ontology terms, plays as an important role as a negative regulator of mast cell activation in suppressing the inflammation response in vivo [32]. Gene ontology terms related to enzymes activity, including, transferase, acyltransferase, phosphotransferase, and kinase, lipid, phospholipid, and phosphatidylinositol binding, were annotated to the MF category. Results of the current study suggest that resistance to the suppression of immune functions and lipid metabolism induced by HS might contribute to heat tolerance in pigs.

Our co-expression analysis identified multiple modules that were highly correlated with semen quality parameters in heat-tolerant and heat-susceptible pigs during the HS period. Of these modules, 1 out of 4 and 4 out of 7 were significantly enriched with GO terms in heat-tolerant and heat-susceptible pigs, respectively. The regulation of cell adhesion, especially the leukocyte cell adhesion was enriched in the *greenyellow* module, which is negatively correlated (*r* = − 0.88, *P*-value < 0.05) with total sperm. Cell adhesion, achieved via cell junctions, is an important cellular activity involved in spermatogenesis in the testis of animals [33]. Genes with top 10 connectivity values within a given module were identified as hub genes. The gene FAS (Fas cell surface death receptor), is well known to play a key role in germ cell apoptosis, which could result in poor semen quality of ejaculated sperm [34]. The *red* module, which is positively correlated with the total sperm number per ejaculate, while it is negatively correlated with motility, was mostly enriched in RNA polymerase regulation in gene transcription. The *pink* module, was positively correlated with the total sperm while negatively correlated with the rejected ejaculated rate and was enriched in phosphatidylinositol related binding. The intra-module connectivity of genes within each module was calculated to identify hub genes which displayed a large degree of connectivity. The hub genes of the different modules could potentially be candidate genes for selection of pigs with unaffected semen quality parameters during HS. However, further research would be needed to confirm the results of the current study given the small sample size employed here. Within this study we obtained correlations between groups of genes and semen parameter traits. These results should however be interpreted with caution since causality among the investigated factors cannot be inferred from these data. Further experiments aimed at specifically testing causality hypotheses should be performed. Finally, because of the high sensitivity of semen to overall stressors, it is difficult to completely separate heat from other forms of stress, such as restraint stress, noise, and social stress. In the current study we tried our best to minimize any potential confounding due to these additional factors through experimental design and similar handling of all the boars, yet the replicate of the current work would be necessary to eliminate the possibility of confounding from other forms of stress.

## Conclusions

In this study, semen quality parameters, including total sperm number per ejaculate, motility, normal morphology rate, droplets, and rejected ejaculate rate, were employed as indicator traits of HS response. Changes of these parameters from TH to HS period were used to classify pigs into heat-tolerant and heat-susceptible groups. Differential gene expression analysis and WGCNA were performed and provided new insights into individual genes and gene networks that are related to HS response. We found changes in transcript expression profile in the heat-tolerant group from the TN to the HS period, and several genes were differentially expressed in the heat-tolerant group compared to the heat-susceptible group under HS. Moreover, different co-expression patterns were detected between the heat-tolerant and heat-susceptible groups during the HS period. Several DEGs and hub genes contained within network modules were associated with immune activities in the heat-tolerant group. The findings in the present study contribute to the development of biomarkers for heat tolerance ability, as well as a better understanding of the biological mechanisms underlying heat tolerance in pigs.

## Methods

### Animals and data collection

Mature unrelated crossbred boars (26 + 2 months old; 237 + 7 kg, *n* = 12) from a three-breed rotational crossbreeding system including Duroc, Hampshire, and Spots breeds were used in the study. Boars came from the North Carolina State University Swine Education Unit (Raleigh, NC, USA) and after completion of the study they were kept for reproductive, teaching, and research purposes at the same unit. All experimental procedures performed on the boars were approved by the North Carolina State University Institutional Animal Care and Use Committee (NCSU 15–115-A). Boars were classified a priori as being either heat-tolerant or heat-susceptible in the following manner.

Weekly sperm production data, including total sperm number per ejaculate, sperm motility, normal morphology rate, cytoplasmic droplets, and rejected ejaculated rate, collected when the boars were between 14 and 25 months of age were used to classify boars into heat-tolerant and heat-susceptible groups for two periods based on normal climatic conditions for the southeastern U.S. in 2016: January through May (TN) and July through October (HS) in Raleigh, NC, USA. During the TN period, the average, minimum, and maximum temperatures were 11 °C, − 14 °C, and 32 °C, respectively. In contrast, the average, minimum, and maximum temperatures were 26 °C, 0 °C, and 36 °C, respectively during the HS period. In July and August, the average daily high temperature was above 26 °C of 62 days.

Semen from all boars was collected by a single experienced technician using the double-gloved technique [35] with powder-free polyvinyl gloves (IMV America, Eden Prairie, MN) into a plastic thermos pre-warmed to 37 °C lined with a plastic collection bag (Minitube of America, Verona, WI). The gel fraction and other contaminants were filtered out of the sample using a milk filter (IMV International, Eden Prairie, MN) placed on top of the thermos and secured with a rubber band. For each ejaculate total sperm were determined by multiplying the collection volume (mL) by sperm concentration which was determined using a SpermaCue® (Minitube of America, Verona, WI). Ejaculates were then transported 7 miles to an on-campus laboratory where motility and morphology data were obtained using a phase contrast microscope (BMX-41, Olympus, Arlington, VA) equipped with a digital video camera (Minitube of America, Verona, WI) and computer-assisted-sperm-analysis software (SpermVision®; Minitube of America, Verona, WI) as previously described [36].

According to the semen quality data collected in TN and HS periods in 2016, boars were classified into heat-tolerant and heat-susceptible groups. Blood samples used for RNA sequencing and their corresponding semen quality parameters analyzed in this study were collected together from the same groups of boars in the middle of February (TN) and at the end of July (HS) in 2017. The average temperature in TN period was 2 °C, and in HS period was 26 °C, respectively. An overall picture of the experimental design is shown in Additional file 1A.

### RNA isolation and sequencing

Blood (6 mL) was taken from a marginal ear vein from each boar during collection with a 23 gauge, 2.54 cm attached to a 25.4 cm plastic tubing (1-in. Infusion kit, B&D, Chicago, IL, USA). Samples were stored in dry ice and transported immediately back to the on-campus laboratory where white blood cells were isolated and stored at -80 °C. RNA was extracted from the blood samples using the Direct-zol™ RNA MiniPrep kit according to the manufacturer’s protocol. Illumina RNA library construction and sequencing were performed in the North Carolina State Genomics Sciences Laboratory (Raleigh, NC, USA). Integrity, purity, and concentration of RNA were checked using an Agilent 2100 Bioanalyzer with an RNA 6000 Nano Chip (Agilent Technologies, Santa Clara, CA, USA). Complementary DNA (cDNA) libraries were constructed using the NEBNext Ultra Directional RNA Library Prep Kit (NEB) and NEBNext Multiplex Oligos for Illumina (NEB) according to the manufacturer’s protocol. Clustering and sequencing of quantified libraries were performed on an Illumina HiSeq 2500 DNA sequencer using a 125 bp single end sequencing reagent kit (Illumina, San Diego, CA, USA).

### Differential expression analysis

Quality control was conducted on RNA-seq reads files using FastQC (v.0.10.1) [37]. Reads were aligned to the swine reference genome assembly (Sus_scrofa.11.1) using TopHat2 (v.2.0.14) [38], with the gene annotation file (Sus_scrofa.Sscrofa11.1.94.gtf) providing additional information from the Ensembl database. Overall alignment rate was 76% across samples. The average of total reads processed was 33,977,416 across samples, of which 25,759,021 were uniquely mapped. Gene counts were computed by featureCounts from Subread (v.1.6.3) [39]. A total of 25,880 genes were found as raw counts. Counts with counts per million (CPM) above 1 in at least 5 samples were kept across samples. Final counts were normalized using the trimmed means method with the ‘*edgeR*’ package (v.3.26.8) in R [40].

The package ‘*limma*’ (v.3.40.6) was used to screen differentially expressed genes (DEGs) between heat-tolerant and heat-susceptible pigs in TN and HS periods [20]. The following model was fitted in the analysis:

$$ {y}_{ij kl}=\upmu +{P}_i+{S}_j+{PS}_{ij}+{B}_k+{\varepsilon}_{ij kl} $$

Where *y*_*ijkl*_ is the raw number of gene counts; μ  is the overall intercept of gene counts; *P*_*i*_ is the fixed effect of the ith class of period (i = TN, HS); *S*_*j*_ is the fixed effect of the jth class of tolerance class (j = susceptible, tolerant); *PS*_*ij*_ is the interaction effect of period and tolerance class; *B*_*k*_ is the random effect of the kth class of boar; *ε*_*ijkl*_ is the random residual. Vectors for the random effects were assumed normally and independently distributed with mean equal to 0 and variance equal to the estimated variances ${\sigma}_b^2$ and ${\sigma}_e^2$, respectively.

Multiple test correction was performed by applying the Benjamini-Hochberg method [41] to the *p*-values based on the number of genes under each treatment condition to control the FDR. Genes with false discovery rate (FDR) < 0.05 were selected as DEGs for interpretation and following analysis.

### Weighed gene co-expression network analysis (WGCNA)

The co-expression scale-free network was built on each experimental group individually using the *“WGCNA”* package (v.1.68) in R language [42]. Data processing was the same as described previously in the differential expression analysis. Normalized data was transformed into reads per kilobase of transcripts per million-mapped to minimize the effect of gene length bias when relating expression levels across genes as described in the WGCNA manual [42]. The data were log-transformed using *log*_*2*_*(x + 1)*. Gene co-expression networks were constructed individually on all of genes passed the filtering of heat-tolerant and heat-susceptible pigs under the HS effects to demonstrate a complete picture of expression relationship between genes during HS period, as well as the associations between gene expression and semen quality parameters. An adjacency matrix was created by calculating Pearson’s correlations between each pair of genes. Soft-thresholding power values were determined using the gradient method with a scale-free topology criterion (R^2^) of 0.80. The adjacency matrix was used to construct the topological overlap-based dissimilarity matrix (TOM) and corresponding dissimilarity (1-TOM). Modules of genes were identified using gene hierarchical cluster based on the TOM. The DynamicTree Cut algorithm [43] was used to determine the clusters of highly co-expressed genes. Modules were assigned different colors. Z-scores were used to assess module conservation. The module eigengenes were computed for each module and the first principal gene in the module was defined as the module eigengene (ME) capturing the maximal amount of variation of each module. Modules highly correlated (r > 0.80) were further merged into a single module. Based on the identified modules, correlations between module eigengenes and sperm quality parameters were estimated. The absolute values of correlation greater than 0.50 with a significance level less than 0.10 were considered as the criteria to select modules for further investigation. In addition, selected modules containing DEGs were kept for gene annotation and enrichment analyses. Module membership measured by module eigengenes was used to quantify the relationship of a gene to a given module. As the interaction of a gene with all other genes in the module, the connectivity of all genes in selected modules was estimated using the *intramoduleConnectivity* function in WGCNA package (v.1.68) [42]. Genes within the selected modules were ranked based on connectivity and top-ranking genes were considered as hub genes within a given module.

### Functional enrichment analysis

Genes annotation was performed using the ‘*org. Ss.eg.db*’ database in the ‘*biomaRt*’ package (version 2.40.4) in R [44]. Gene symbols and ENTREZ gene IDs were annotated. Genes of interest (DEGs and genes within modules of interest) were fed to gene ontology (GO) enrichment analysis using the *enrichGO* function of ‘*clusterProfiler*’ package (v.3.12.0) in R [45]. The FDR < 0.05 was used as the cut-off threshold. An overall view of RNA-seq data processing and gene expression analysis processes is presented in Additional file 1B.

## Supplementary information

**Additional file 1.** Experimental design and RNA-seq analysis pipeline. This figure presents the experimental design (A) and gene expression analysis pipeline (B).

**Additional file 2.** Gene dendrogram of the co-expression networks. This figure presents the number of modules with colors and the clustering relationship between models identified in the co-expression networks constructed in heat-tolerant pigs under HS (A) and heat-susceptible pigs under HS (B).

**Additional file 3.** Association between Module Membership of modules and the significance of DEGs within each module. This figure presents the roles of DEGs in each selected models in terms of Module Membership in heat-tolerant pigs under HS (A) and heat-susceptible pigs under HS (C). Descriptive statistics table of modules was included (C & D).

**Additional file 4.** Mean of semen quality parameters by heat-tolerant and heat-susceptible groups across TN and HS periods. This figure presents the changes in semen quality parameters of boars measured in 2016 and 2017. Total sperm number per ejaculate (A), motility (B), normal morphology rate (C), droplets (D), rejected ejaculate rate (E).

## Data Availability

The datasets supporting the conclusions of this article are included within the article and its additional files.

## Abbreviations

HS: Heat stress
TN: Thermoneutral
CPM: Counts per million
DEGs: Differentially expressed genes
FDR: False discovery rate
WGCNA: Weighted gene co-expression network analysis
TOM: Topological overlap-based dissimilarity matrix
ME: Module eigengene
GO: Gene ontology
BP: Biological process
MF: Molecular function
CC: Cellular component
MM: Module membership
GS: Gene significance
