# Supplementary material for: Transcriptome analysis identifies genes and co-expression networks underlying heat tolerance in pigs
Source: BMC Genet. 2020 Apr 21;21:44. doi: 10.1186/s12863-020-00852-4 (PMC7171765; doi:10.1186/s12863-020-00852-4)
Supplement: Supplementary file 1 — Additional file 1. Experimental design and RNA-seq analysis pipeline. This figure presents the experimental design (A) and gene expression analysis pipeline (B). [file 12863_2020_852_MOESM1_ESM.pdf]

A

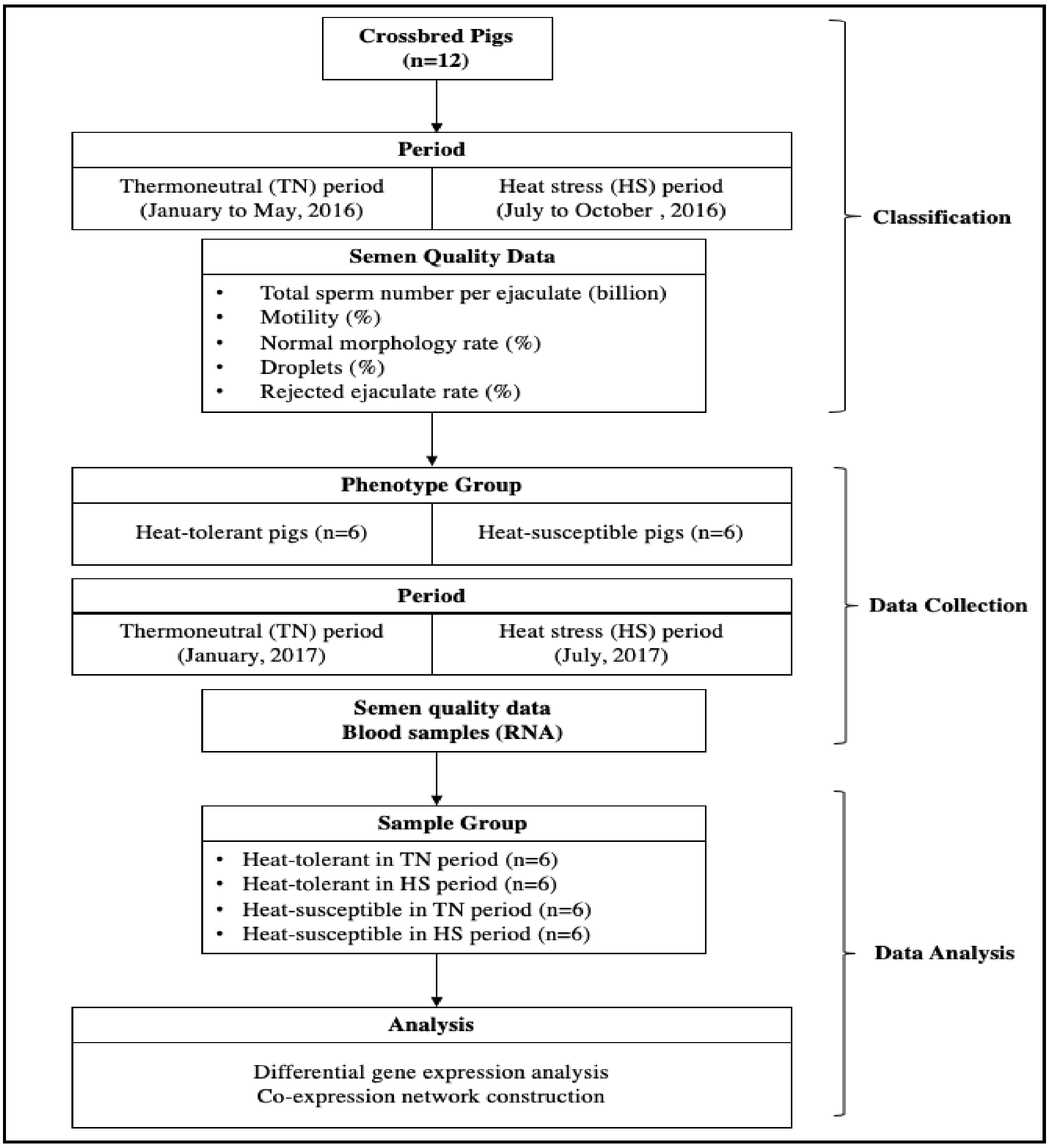
Crossbred Pigs
(n=12)
Period
Thermoneutral (TN) period
(January to May, 2016)
Heat stress (HS) period
(July to October , 2016)
Semen Quality Data
Total sperm number per ejaculate (billion)
Motility (%)
Normal morphology rate (%)
Droplets (%)
Rejected ejaculate rate (%)
Classification
Phenotype Group
Heat-tolerant pigs (n=6)
Heat-susceptible pigs (n=6)
Period
Thermoneutral (TN) period
(January, 2017)
Heat stress (HS) period
(July, 2017)
Semen quality data
Blood samples (RNA)
Data Collection
Sample Group
Heat-tolerant in TN period (n=6)
Heat-tolerant in HS period (n=6)
Heat-susceptible in TN period (n=6)
Heat-susceptible in HS period (n=6)
Analysis
Differential gene expression analysis
Co-expression network construction
Data Analysis

B

RNA-seq Analysis Pipeline

**Processes:**	**Tools:**
• QC of Raw reads(.fastq)<br>• Read Alignment (.bam)<br>• Raw gene counts (.txt)	• FastQC (v.0.10.1)<br>• TopHat2 (v.2.0.14)<br>• featureCounts (v.1.6.3)

**Differential Gene Expression Analysis** (edgeR and Limma)	**Co-expression Network Analysis** (edgeR and WGCNA)
**Data Processing:**<br>• Raw count (genes = 25880)<br>• Remove low count (genes = 10761)<br>• Normalization using Trimmed Means method (TMM)<br>• Transformed to counts per million (CPM)<br><br>**Data Analysis:**<br>• To_HS vs. To_TN<br>• Su_ HS vs. Su_TN<br>• To_HS vs. Su_HS<br>• To_TN vs. Su_TN<br><br>**Gene Annotation:**<br>• KEGG pathway mapping<br>• GO term enrichment analysis	**Data Processing:**<br>• Raw count (genes = 25880)<br>• Remove low count (genes = 10761)<br>• Normalization using Trimmed Means method (TMM)<br>• Transformed to reads per kilobase of transcripts per million-mapped (RPKM)<br>• Transformed to log2<br><br>**Data Analysis:**<br>• Module detection within To_HS<br>• Module detection within Su_HS<br>• Correlation with semen quality parameters<br><br>**Gene Annotation:**<br>• KEGG pathway mapping<br>• GO term enrichment analysis

Results

- Differentially expressed genes (DEGs)
- Network modules with hub genes
- Relationships between modules and semen quality parameters
- Functional enrichment terms of DEGs and genes within modules
- KEGG pathways associated with DEGs and genes within modules
