# Supplementary figures and images for: Transcriptome analysis identifies genes and co-expression networks underlying heat tolerance in pigs
Source: BMC Genet. 2020 Apr 21;21:44. doi: 10.1186/s12863-020-00852-4 (PMC7171765; doi:10.1186/s12863-020-00852-4)

**A**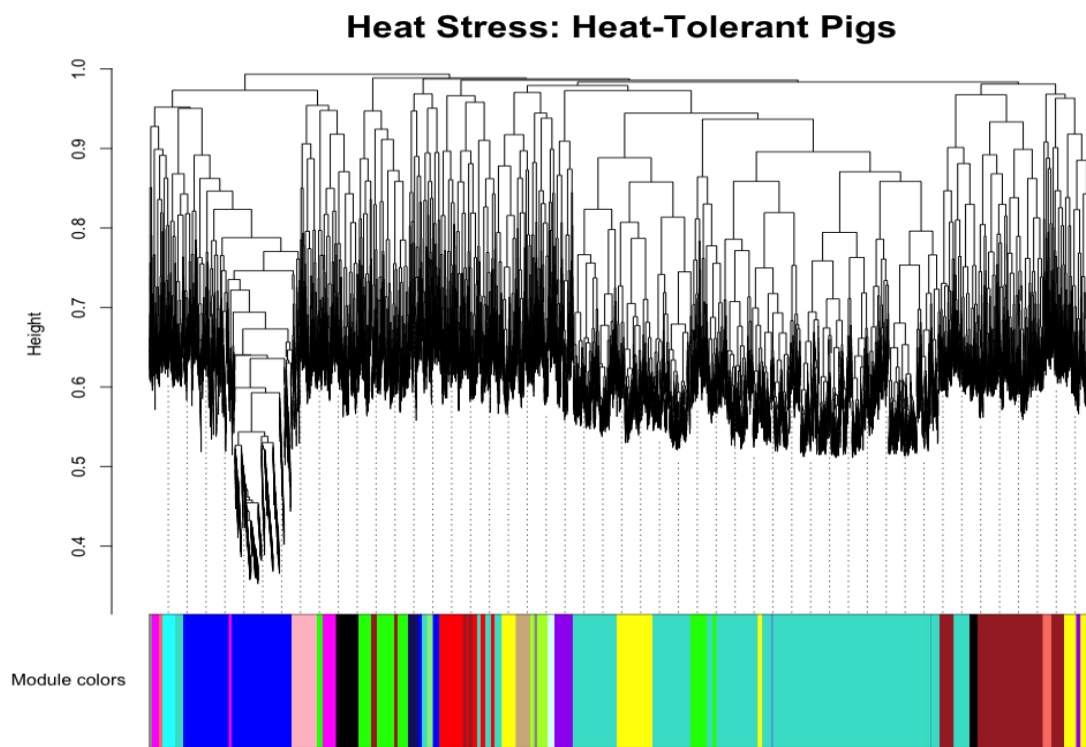**B**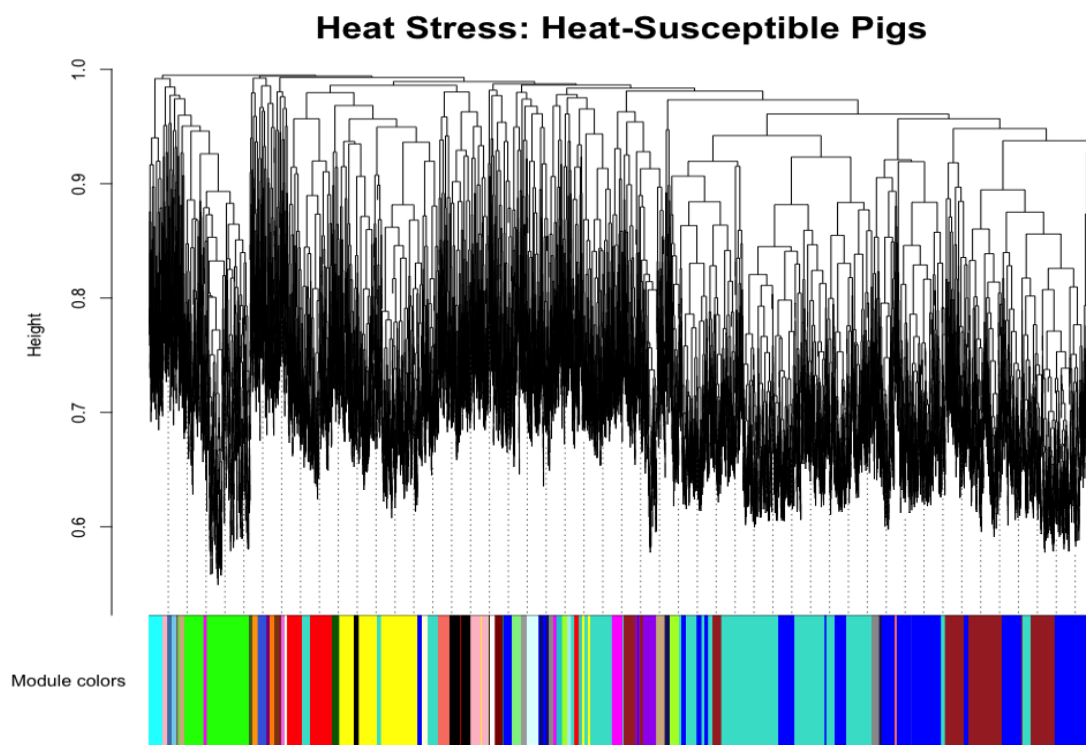

Supplement: Supplementary file 2 — Additional file 2. Gene dendrogram of the co-expression networks. This figure presents the number of modules with colors and the clustering relationship between models identified in the co-expression networks constructed in heat-tolerant pigs under HS (A) and heat-susceptible pigs under HS (B). [file 12863_2020_852_MOESM2_ESM.pdf]

**A**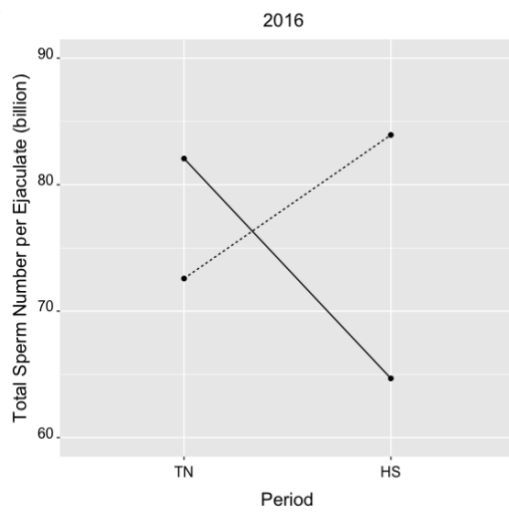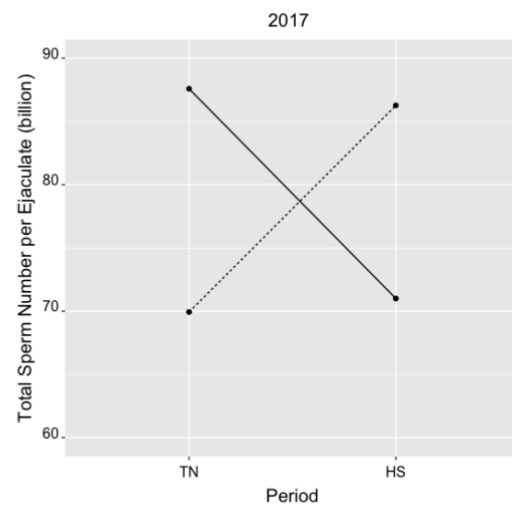**B**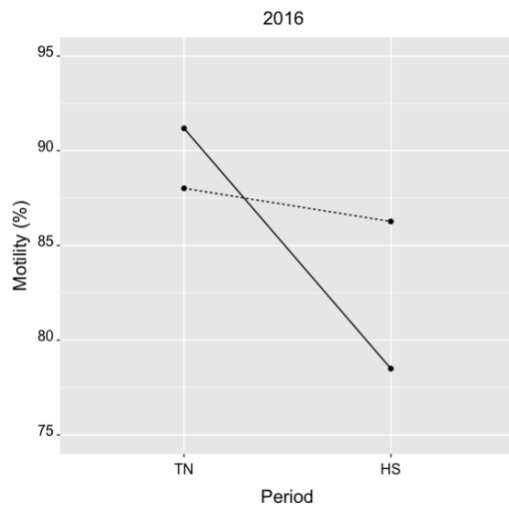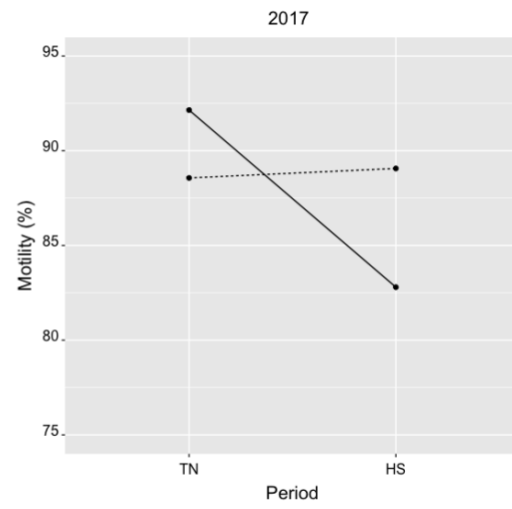

**C**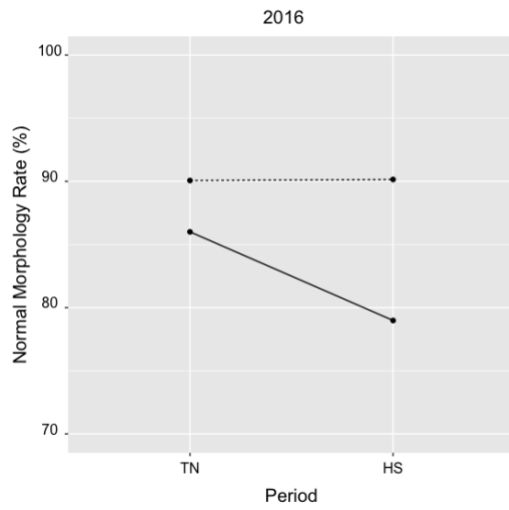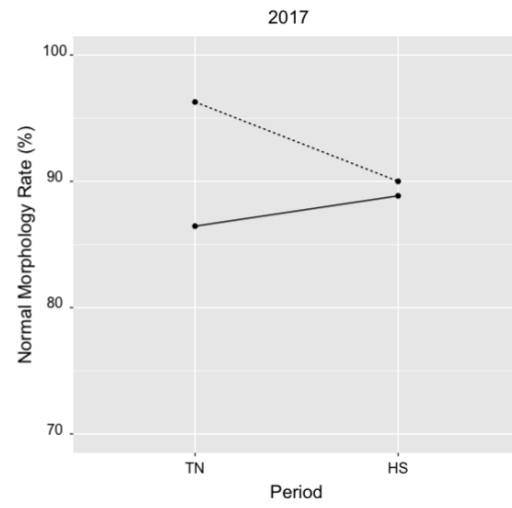**D**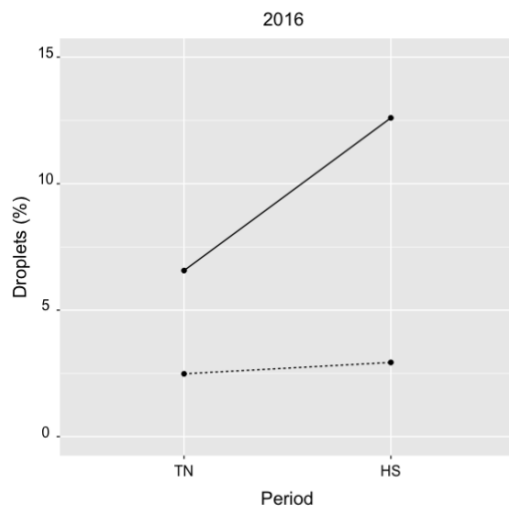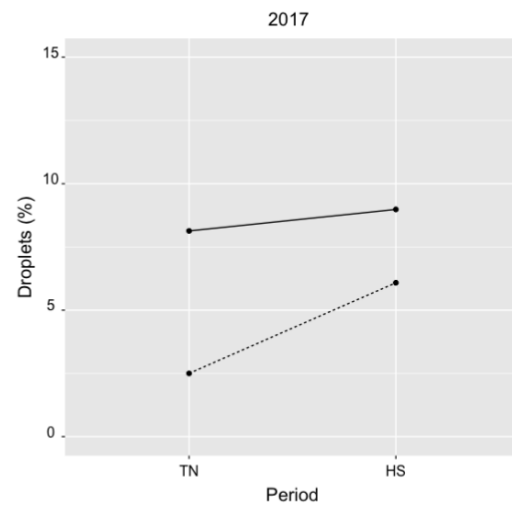**E**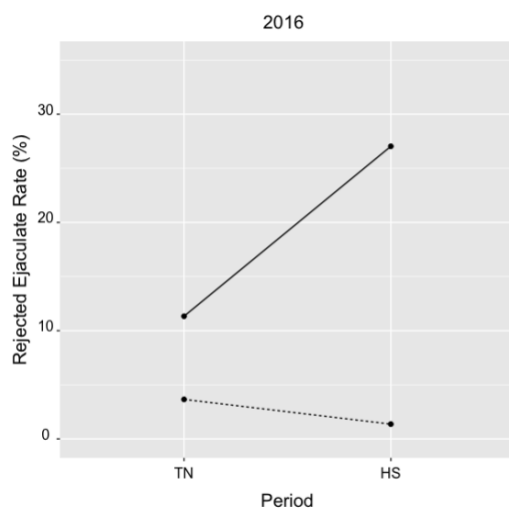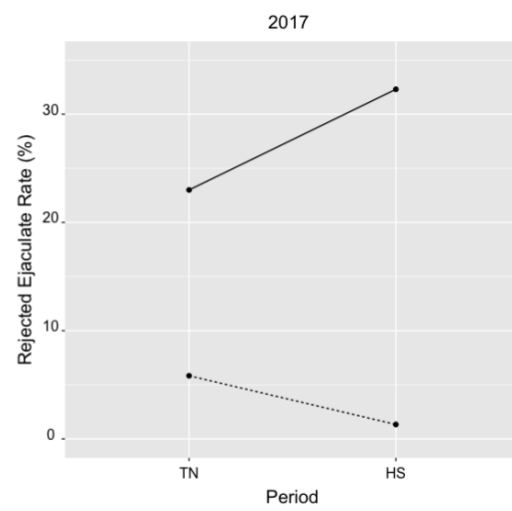

Supplement: Supplementary file 4 — Additional file 4. Mean of semen quality parameters by heat-tolerant and heat-susceptible groups across TN and HS periods. This figure presents the changes in semen quality parameters of boars measured in 2016 and 2017. Total sperm number per ejaculate (A), motility (B), normal morphology rate (C), droplets (D), rejected ejaculate rate (E). [file 12863_2020_852_MOESM4_ESM.pdf]
