# Supplementary material for: Transcriptome analysis identifies genes and co-expression networks underlying heat tolerance in pigs
Source: BMC Genet. 2020 Apr 21;21:44. doi: 10.1186/s12863-020-00852-4 (PMC7171765; doi:10.1186/s12863-020-00852-4)
Supplement: Supplementary file 3 — Additional file 3. Association between Module Membership of modules and the significance of DEGs within each module. This figure presents the roles of DEGs in each selected models in terms of Module Membership in heat-tolerant pigs under HS (A) and heat-susceptible pigs under HS (C). Descriptive statistics table of modules was included (C & D). [file 12863_2020_852_MOESM3_ESM.pdf]

A

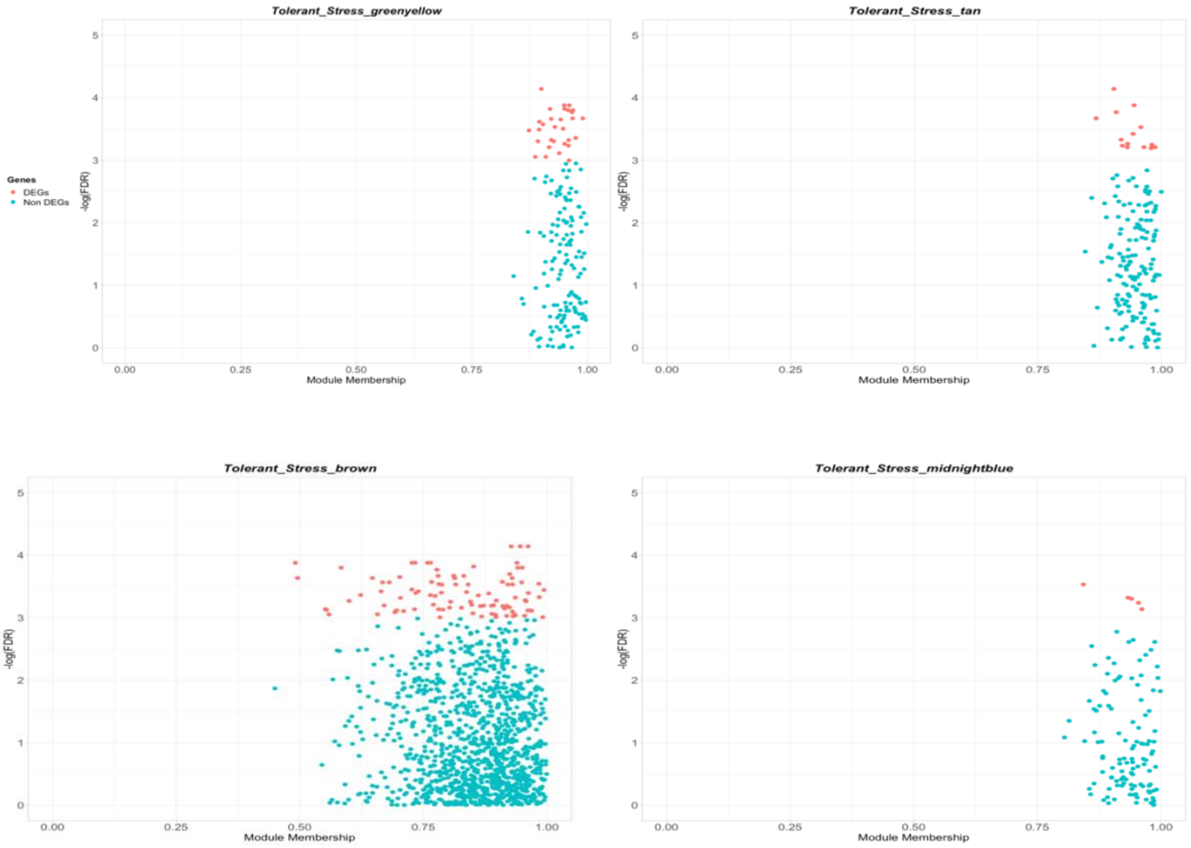

B

Module	Semen Parameter	Correlation	P-value	Genes	DEGs
Greenyellow	Total Sperm	-0.88	0.05	168	30
Tan	Motility	0.88	0.05	167	14
Midnightblue	Normal Morphology	-0.93	0.02	120	5
	Droplets	0.93	0.02		
Brown	Reject Ejaculate	-0.89	0.04	1234	98

C

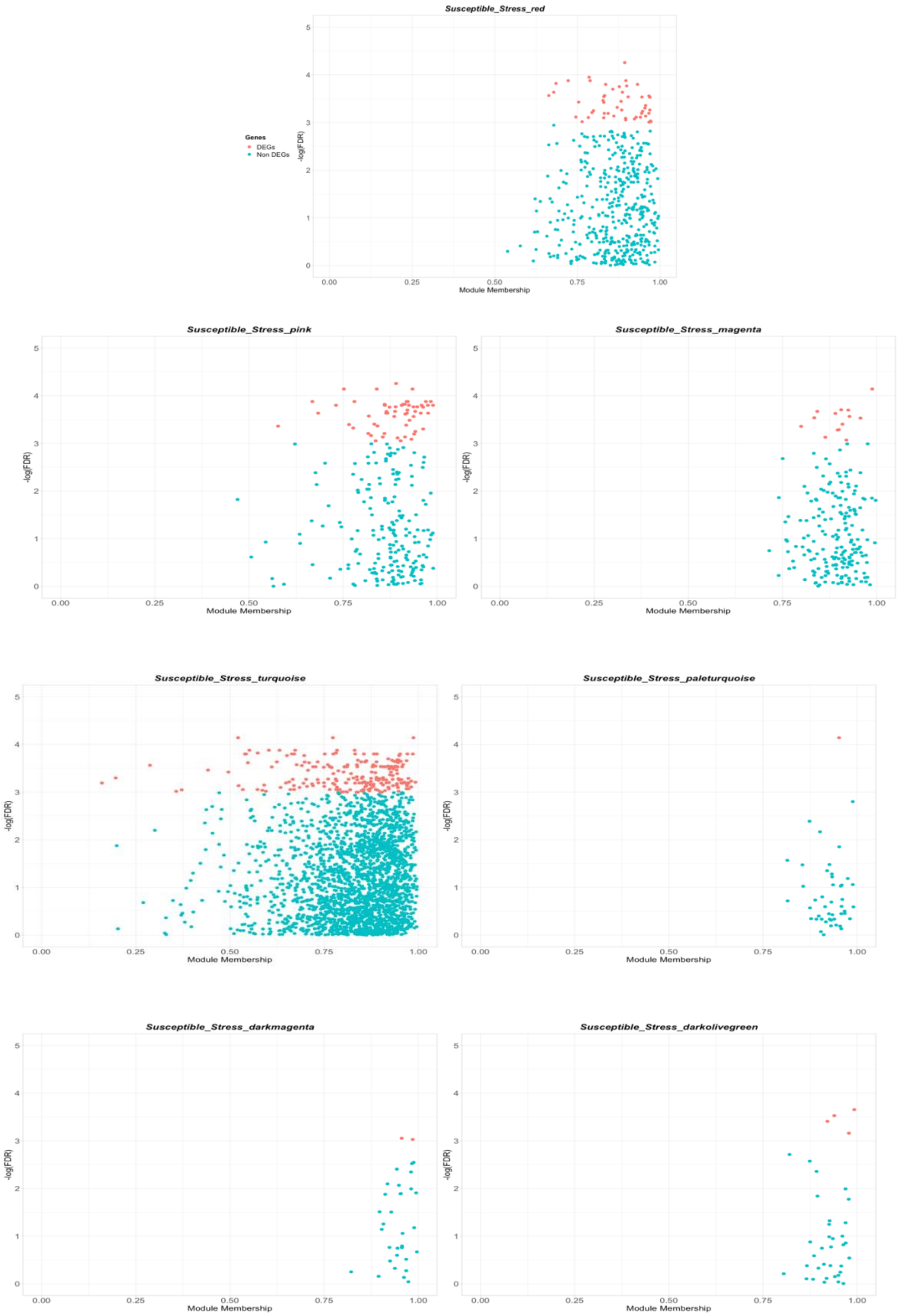

Susceptible_Stress_red
Genes
DEGs
Non DEGs
-log(FDR)
Module Membership
Susceptible_Stress_pink
Susceptible_Stress_magenta
Susceptible_Stress_turquoise
Susceptible_Stress_paleturquoise
Susceptible_Stress_darkmagenta
Susceptible_Stress_darkolivegreen

D

Module	Semen Parameter	Correlation	P-value	Genes	DEGs
Red	Total Sperm<br>Motility	0.87<br>-0.84	0.02<br>0.04	480	51
Pink	Total Sperm<br>Rejected Ejaculate	-0.94<br>0.91	0.005<br>0.01	250	58
Magenta	Motility	-0.88	0.02	199	14
Turquoise	Motility	-0.86	0.03	2380	214
Paleturquoise	Normal Morphology<br>Droplets	0.85<br>-0.84	0.03<br>0.04	46	1
Darkmagenta	Normal Morphology<br>Droplets	0.85<br>-0.81	0.03<br>0.05	33	2
Darkolivegreen	Normal Morphology<br>Droplets	0.92<br>-0.92	0.01<br>0.01	38	4
